# Protein Microarray-Guided Development of a Highly Sensitive and Specific Dipstick Assay for Glanders Serodiagnostics

**DOI:** 10.1128/jcm.01234-22

**Published:** 2022-12-21

**Authors:** Gabriel E. Wagner, Andreas Berner, Michaela Lipp, Christian Kohler, Karoline Assig, Sabine Lichtenegger, Muhammad Saqib, Elke Müller, Trung T. Trinh, Anne-Marie Gad, Hans Hermann Söffing, Ralf Ehricht, Karine Laroucau, Ivo Steinmetz

**Affiliations:** a Diagnostic & Research Institute of Hygiene, Microbiology and Environmental Medicine, Medical University of Graz, Graz, Austria; b Friedrich Loeffler Institute for Medical Microbiology, Greifswald, Germany; c Department of Clinical Medicine and Surgery, University of Agriculture, Faisalabad, Pakistan; d InfectoGnostics Research Campus, Centre for Applied Research, Jena, Germany; e Leibniz-Institute of Photonic Technology (Leibniz-IPHT), Jena, Germany; f Institute of Microbiology and Biotechnology, Vietnam National University, Hanoi, Vietnam; g Senova Gesellschaft für Biowissenschaft und Technik mbH, Weimar, Germany; h Friedrich Schiller University Jena, Institute of Physical Chemistry, Jena, Germany; i Paris Est University, Anses, Animal Health Laboratory, Bacterial zoonosis UnitMaisons-Alfort Cedex, France; Marquette University

**Keywords:** glanders, immune response, diagnostic test, serology, point-of-care test

## Abstract

Burkholderia mallei, the causative agent of glanders, is a clonal descendant of Burkholderia pseudomallei, the causative agent of melioidosis, which has lost its environmental reservoir and has a restricted host range. Despite limitations in terms of sensitivity and specificity, complement fixation is still the official diagnostic test for glanders. Therefore, new tools are needed for diagnostics and to study the B. mallei epidemiology. We recently developed a highly sensitive serodiagnostic microarray test for human melioidosis based on the multiplex detection of B. pseudomallei proteins. In this study, we modified our array tests by using anti-horse IgG conjugate and tested sera from B. mallei-infected horses (*n* = 30), negative controls (*n* = 39), and horses infected with other pathogens (*n* = 14). Our array results show a sensitivity of 96.7% (confidence interval [CI] 85.5 to 99.6%) and a specificity of 100.0% (CI, 95.4 to 100.0%). The reactivity pattern of the positive sera on our array test allowed us to identify a set of 12 highly reactive proteins of interest for glanders diagnosis. The B. mallei variants of the three best protein candidates were selected for the development of a novel dipstick assay. Our point-of-care test detected glanders cases in less than 15 min with a sensitivity of 90.0% (CI, 75.7 to 97.1%) and a specificity of 100.0% (CI, 95.4 to 100.0%). The microarray and dipstick can easily be adopted for the diagnosis of both B. mallei and B. pseudomallei infections in different animals. Future studies will show whether multiplex serological testing has the potential to differentiate between these pathogens.

## INTRODUCTION

Glanders is a contagious disease of Equidae, such as horses and donkeys, which can also be transmitted to humans. It was first reported by Hippocrates in around 425 BC and described around 100 years later by Aristoteles, who named the disease “melis.” Glanders is caused by Burkholderia mallei, an immotile, facultative, intracellular bacterium closely related to Burkholderia pseudomallei, the causative agent of melioidosis (1, 2). Both bacteria are classified as tier 1 agents (potential bioweapons) by the U.S. Federal Select Agents Program, with B. mallei having a long history in warfare.

The pathogen B. mallei is highly adapted to its host and cannot survive in the environment for an extended period. Therefore, B. mallei is usually transmitted through contaminated food or water (3, 4). In addition, the disease can be acquired through the inhalation of contagious aerosols, skin abrasions, or mucous membranes (5). The incubation period ranges from 6 days to several months, which complicates tracing the source of infection (6). The disease presents mainly in its acute form in donkeys and in its chronic form in horses (7, 8).

B. mallei was eradicated from western Europe, Canada, Australia, and North America through stringent eradication programs but is still endemic in North Africa, South America, the Middle East, and Asia (9, 10). Nevertheless, the international trade and movement of animals in combination with only scarce epidemiology data on B. mallei prevalence and poorly standardized diagnostic tools could lead to the reintroduction of glanders into B. mallei-free regions (11). Indeed, over the past few decades the number of outbreaks has increased again, rendering glanders a reemerging disease of priority to animal health authorities (12, 13).

The complement fixation test (CFT) is the serological test prescribed by the World Organization for Animal Health for the international trade of equines, although the sensitivity and specificity of the CFT can vary. Indeed, depending on the antigen used and the method in place (14, 15), the sensitivity of this test can range between 62.5 and 100.0% (11). The anticomplementary activity of equine sera and false-positive CFT results are further limitations (14, 15). Alternative tests (Western blot, enzyme-linked immunosorbent assays [ELISAs], Luminex) have been developed, and recent validation works give hope for improving the glanders diagnostic performance (16–18). In particular, the use of recombinant proteins (including Hcp1 and GroEL) has recently led to promising prospects for glanders diagnosis (17, 19, 20). However, additional antigens might be useful to increase the performance of serodiagnostic glanders tests and distinguish between exposure and disease, acute and chronic forms, or melioidosis and glanders. Furthermore, a rapid point-of-care glanders test for on-site testing would be highly beneficial for epidemiological purposes and to prevent the spread and reintroduction of the pathogen.

In a previous study, we developed a protein microarray for the multiplex detection of anti-B. pseudomallei antibodies against 20 B. pseudomallei proteins (21). We showed that our array is able to address issues of poor standardization and slow, labor-intensive workflows in the case of B. pseudomallei (21) and allows for the development of a rapid point-of-care assay (22). We hypothesized that it is also capable of detecting anti-B. mallei antibodies, due to the close evolutionary relationship of the two pathogens.

Therefore, the objectives of this study were to (i) evaluate the melioidosis array regarding its serodiagnostic performance for glanders detection and (ii) assess whether B. mallei variants of diagnostic proteins for melioidosis could also be pertinent to develop a quick screening test for equine glanders.

## MATERIALS AND METHODS

### Serum samples.

The following three panels of equid sera were included in this study: P1, 30 positive sera from clinically confirmed glanders cases from Pakistan, where the disease is endemic; P2, 14 control sera of horses infected with pathogens other than B. mallei (dourine [*n* = 3], equine metritis [*n* = 5], equine infectious anemia [*n* = 2], and viral arthritis [*n* = 4]), provided by the European Reference Laboratory for Equine Diseases; and P3, 39 sera of healthy controls obtained from officially glanders-free countries (France [*n* = 7], Ireland [*n* = 3], and Tunisia [*n* = 29]). In agreement with previous studies, horses showing clinical signs related to glanders were regarded glanders cases if they were positive in a serological assay (CFT, indirect ELISA [iELISA]) or in close contact with glanders-positive horses during culture-confirmed outbreaks (19, 20). No ethical approval was required for this study because field sera from the P1, P2 and P3 panels were collected for monitoring and diagnostic purposes.

Sera were analyzed with the CFT, as reported previously (23), and with the ID Screen glanders indirect ELISA (IdVet, France) (19). Regarding the iELISA, results were expressed as S/P = (sample – negative control)/(positive control – negative control) · 100, and samples were considered positive if the S/P value was >50 and undetermined if 40 < S/P ≥ 50. The data are summarized in Table S1 in the supplemental material. Of note, iELISA results of some sera were already acquired for our previous study and were therefore published before (17). These iELISA results are indicated by red type in Table S1. Results were used for a method comparison in the present study.

### Protein microarray-based analysis.

Our previously published B. pseudomallei protein microarray containing 20 B. pseudomallei antigens (21) was supplemented by two additional proteins (BPSS1498 and BPSS1840) in this study. The DNA sequences of the two proteins were amplified by PCR using B. pseudomallei K96243 genomic DNA as a template and the primers listed in Table S2. The fragments obtained were cloned into the respective expression vectors (Table S2) and verified by Sanger sequencing. BPSS1498 was expressed and purified as described for the Strep-tagged proteins in reference 21.

The expression vector of BPSS1840 was transformed into Escherichia coli BL21(DE3)pLysS cells (Promega, Germany) by heat shock. A volume of 1 L of LB medium was inoculated with 10 mL of overnight culture, and cells were grown to an optical density at 600 nm (OD_600_) of 0.6 to 0.8 at 37°C. At this point, protein expression was induced by the addition of isopropyl-β-d-thiogalactopyranoside (Carl Roth, Germany) at a final concentration of 1 mM and carried out at 20°C overnight. Subsequently, cells were harvested by centrifugation (4,000 × *g*, 4°C, 30 min) and disrupted by sonication (15 min, 0.5 s on/off cycle, 90% amplitude on a Sonoplus HD 2070 [Bandelin, Germany]). The lysates were centrifuged at 10,000 × *g* at 4°C for 1 h to remove debris and insoluble compounds. The recombinant protein was purified from the supernatant using a gravity flow Ni-NTA column (Qiagen, Germany) according to the manufacturer’s instructions. The tag of the purified protein was removed by a tobacco etch virus protease cleavage step at 4°C overnight, followed by a reversed Ni-NTA purification step, where the cleaved tag-free protein was eluted in the flowthrough. Finally, the protein antigen was dialyzed against Dulbecco’s phosphate-buffered saline (Gibco-Life Technologies, USA) and stored at −20°C until use.

The protein microarray was constructed as described previously (21). Information about the protein antigen constructs and their homology to the closest protein match in B. mallei are summarized in Table 1.

**TABLE 1:** Burkholderia pseudomallei antigens used for the construction of the protein microarrays and their Burkholderia mallei homologs

No.	Locus^*a*^	Definition^*a*^	Expressed^*b*^	Identity [*n* (%)]^*c*^
1	BPSL0280	Flagellar hook-associated protein FlgK	All 667 aa	667/714 (93.4)
2	BPSL1445	Putative lipoprotein	aa 23–195	195/195 (100.0)
3	BPSL1661	Putative hemolysin-related protein	aa 1–683	No similarity
4	BPSL1661	Putative hemolysin-related protein	aa 1001–2000	No similarity
5	BPSL2030	Putative exported protein	aa 23–186	185/186 (99.5)
6	BPSL2096	Putative hydroperoxide reductase	All 182 aa	182/182 (100.0)
7	BPSL2520	Putative exported protein	aa 22-198	198/200 (99.0)
8	BPSL2522	Outer membrane protein a	aa 23–224	224/224 (100.0)
9	BPSL2697	Molecular chaperone GroEL 1	All 546 aa	544/552 (98.6)
10	BPSL2698	Cochaperonin GroES 1	All 97 aa	97/97 (100.0)
11	BPSL3319	Flagellin FliC	All 388 aa	388/388 (100.0)
12	BPSS0476	Cochaperonin GroES 2	All 96 aa	76/97 (78.4)
13	BPSS0477	Molecular chaperone GroEL 2	All 546 aa	460/552 (83.3)
14	BPSS0530	Hypothetical protein ImpJ	All 453 aa	244/453 (53.9)
15	BPSS1385	Putative ATP/GTP-binding protein	All 328 aa	No similarity
16	BPSS1516	BopC	All 469 aa	448/544 (82.4)
17	BPSS1525	Guanine nucleotide exchange factor BopE	aa 79–261	260/261 (99.6)
18	BPSS1532	Translocator protein BipB	aa 1–344	620/620 (100.0)
19	BPSS1722	Malate dehydrogenase	All 327 aa	327/327 (100.0)
20	BPSS2141	Putative periplasmic oligopeptide-binding protein	aa 40–554	553/554 (99.8)
21	BPSS1498	Hemolysin-coregulated protein 1	All 169 aa	168/169 (99.4)
22	BPSS1840	Putative *N*-acetylmuramoyl-l-alanine amidase	All 344 aa	344/346 (99.4)

a The locus name and definition were obtained from the Kyoto Encyclopedia of Genes and Genomes (www.genome.jp/kegg/) for B. pseudomallei strain K96243.

b The construct length of the antigen expressed. aa, amino acids.

c The identity of B. pseudomallei proteins to B. mallei homologs was obtained by conducting a BLAST search of the B. pseudomallei protein sequence against B. mallei (TaxID 13373). Protein sequences of the best hit were aligned using EMBOSS Needle Alignment (30) against the original B. pseudomallei protein sequence to obtain the identity scores.

The protein microarrays were performed as previously described (21), but the detection conjugate was replaced with an anti-horse IgG-horseradish peroxidase (HRP) antibody. Briefly, arrays were prewashed with 150 μL of array buffer (1× phosphate-buffered saline [PBS], 0.05% Tween 20, 0.25% Triton) for 5 min and then blocked with 100 μL of array buffer supplemented with 2% powdered milk (Carl Roth, Germany) at 37°C and 300 rpm for 5 min. They were subsequently incubated for 30 min at 37°C and 300 rpm with 100 μL of a 1:2,000 dilution of animal serum in array buffer. After one wash, as described above (37°C, 400 rpm, 5 min), the protein arrays were incubated for 30 min at 37°C and 300 rpm with 100 μL of a 1:1,000 HRP-conjugated goat anti-horse IgG antibody (Jackson ImmunoResearch, UK) diluted in array buffer. Protein arrays were washed again twice with washing buffer (37°C, 400 rpm, 4 min) and, finally, incubated with 100 μL of detection substrate D1 (Alere Technologies GmbH, Germany) for 10 min at room temperature without shaking. Arrays were analyzed using the ArrayMate system (Alere Technologies GmbH) after removal of the substrate. Antigen spots were considered positive if the signal intensity was greater than or equal to 0.3.

### Point-of-care (POC) dipstick test.

Based on the results of the array, we chose a set of the three most promising protein antigens for our dipstick assays. The dipstick assay relies on the B. mallei variants of the protein antigens to rule out that slight differences between the B. pseudomallei and B. mallei protein sequence mask specific glanders epitopes. The B. mallei homologues of BPSS1498, BPSL2697, and BPSS1840 are BM45_3167, BM45_901, and BM45_4262, respectively.

These genes were cloned using the primers and expression vectors listed in Table S2 and verified by Sanger sequencing. Genomic DNA of a Vietnamese B. mallei strain, VTCC 70090, served as a template.

The three antigens were expressed and purified as described above for microarray antigen BPSS1840. However, for dipstick assay antigens, an additional fast protein liquid chromatography purification step was applied to minimize nonspecific background signals against E. coli impurities. This previously helped to increase the specificity of our dipstick assays (22). Therefore, BM45_3167 and BM45_4262 were dialyzed against size exclusion chromatography buffer (150 mM NaCl, 50 mM Na_2_HPO_4,_ pH 8), applied to a Hiload 16/600 Superdex 200 pg column (GE Healthcare Bio-Sciences, Germany) and eluted by an isocratic flow of the same buffer. BM45_901 was dialyzed against ion-exchange chromatography buffer (50 mM Tris, 10 mM MgCl_2_, 0.5 mM KCl, 1 mM EDTA, pH 7.5) applied to a Hitrap DEAE FF column (GE Healthcare Bio-Sciences) and eluted with a linear gradient, increasing the NaCl concentration of the buffer from 0 to 1 M. Finally, purified antigens were dialyzed against Dulbecco’s phosphate-buffered saline (Gibco-Life Technologies, USA), concentrated to 2 mg/mL, and stored at −20°C.

Subsequently, antigens were sprayed on nitrocellulose membranes (thickness, 125 to 155 μm; pore size, 10 μm; capillary flow time, 110 to 165 s/4 cm) using a dispenser system (BioDot XYZ-3000 dispensing platform, 1 μL protein solution/cm), and the dipsticks obtained were stored at 4°C until use.

**FIG 1:**
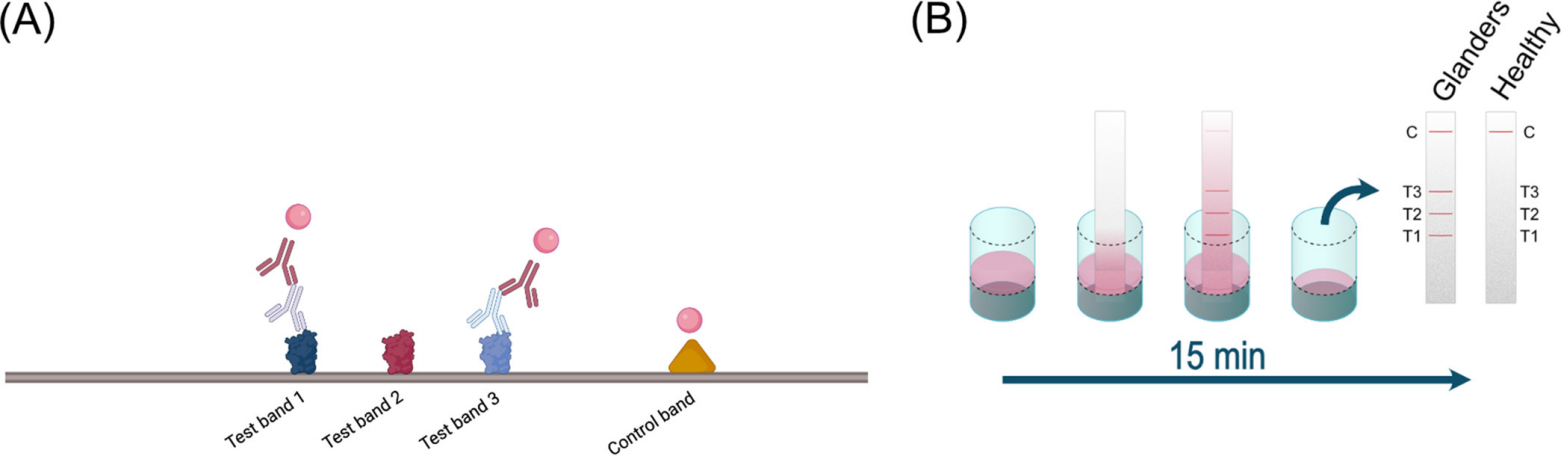
(A) Dipstick test layout and illustration of the indirect ELISA principle used for detection. Figure created with BioRender.com. (B) Testing procedure of the glanders dipstick test developed. An amount of 50 μL of a 1:100 dilution of serum in dipstick master mix is transferred into a well of a microtiter plate. After a 15-min incubation of the dipstick, the strip is removed from the well, and results are read by eye. Up to three lines (T1, T2, and T3, corresponding to the three antigens) plus the control are visible for an infected animal, whereas the presence of the control line alone indicates a negative result. Figure adapted from reference 22.

To detect B. mallei-specific horse IgG antibodies using the dipstick assay, sera were diluted 1:100 in the dipstick master mix comprising the control conjugate (Senova, Germany; diluted 1:50), the gold conjugated goat anti-horse IgG detection antibody (Senova; diluted 1:100), and the running buffer (universal casein diluent buffer Tween; Senova). An amount of 50 μL of the final solution was transferred into a well of a 96-well plate containing the dipstick. After 15 min of incubation, the assay was removed, and band intensities were evaluated by comparing protein bands to the gold reference card (Senova) with scores ranging from 0 (no reaction) to 10 (strong reaction) (see Fig. 1). A cutoff for the readout by eye was defined as 4. All dipsticks were independently evaluated by three individuals. A serum was considered positive if at least one band was observed with any of the three antigens and was considered negative if only the control band was visible.

It is noteworthy that the dipstick included a test band of the BPSS2522 B. mallei homolog. This antigen was hampered by severe background issues and low sensitivity and specificity in preliminary experiments. Therefore, this band was masked and not evaluated in the final assay.

### Statistical analysis and data visualization.

Data analysis and visualization were performed using R v4.0.3 and R-Studio v1.3.1093. Statistical analysis was carried out using SPSS Statistics v25.0.0.1 (IBM, USA). The discriminatory power of each antigen was calculated using Fisher’s two-sided exact test comparing the positive group to both control groups. A *P* value smaller than 0.05 was considered significant. The reported confidence intervals for sensitivities and specificities are Jeffreys intervals. The Cochran’s Q Test for related samples was used to evaluate differences in the diagnostic test performance/method of evaluation. A *P* value equal to or smaller than 0.05 was considered significant. The following formulas were used to calculate diagnostic sensitivities and specificities: sensitivity = ∑ true glanders-positive tested individuals/∑ total glanders-positive individuals; specificity = ∑ true glanders-negative tested individuals/∑ total glanders-negative individuals.

## RESULTS AND DISCUSSION

### A B. pseudomallei antigen microarray shows promise as a well-standardized and fast tool for highly sensitive glanders serodiagnostic and antigen screening.

We developed a highly sensitive and specific protein microarray test for melioidosis in a previous study based on the multiplex detection of the antibody response against 20 B. pseudomallei protein antigens (21). We hypothesized that the close evolutionary relationship between B. pseudomallei and B. mallei (24, 25), nicely exemplified by the high identity scores for most of the antigens utilized (see Table 1), extends the array’s applicability in two interesting and interconnected areas: (i) for glanders serodiagnostics and (ii) to screen for serodiagnostic antigens that might be optimized for specific glanders assays.

An updated version of our microarray-based assay with additional antigens was used for this study. BPSS1498 (hemolysin-coregulated protein 1) was included because of its outstanding performance in melioidosis serology (22, 26, 27), and BPSS1840 (putative *N*-acetylmuramoyl-l-alanine amidase), because of previous in-house screening results.

This new version of the assay was evaluated on a set of positive sera from cases of glanders (P1, *n* = 30), horses suffering from diseases other than glanders (P2, *n* = 14), and negative sera of horses from glanders-free areas (P3, *n* = 39).

**FIG 2:**
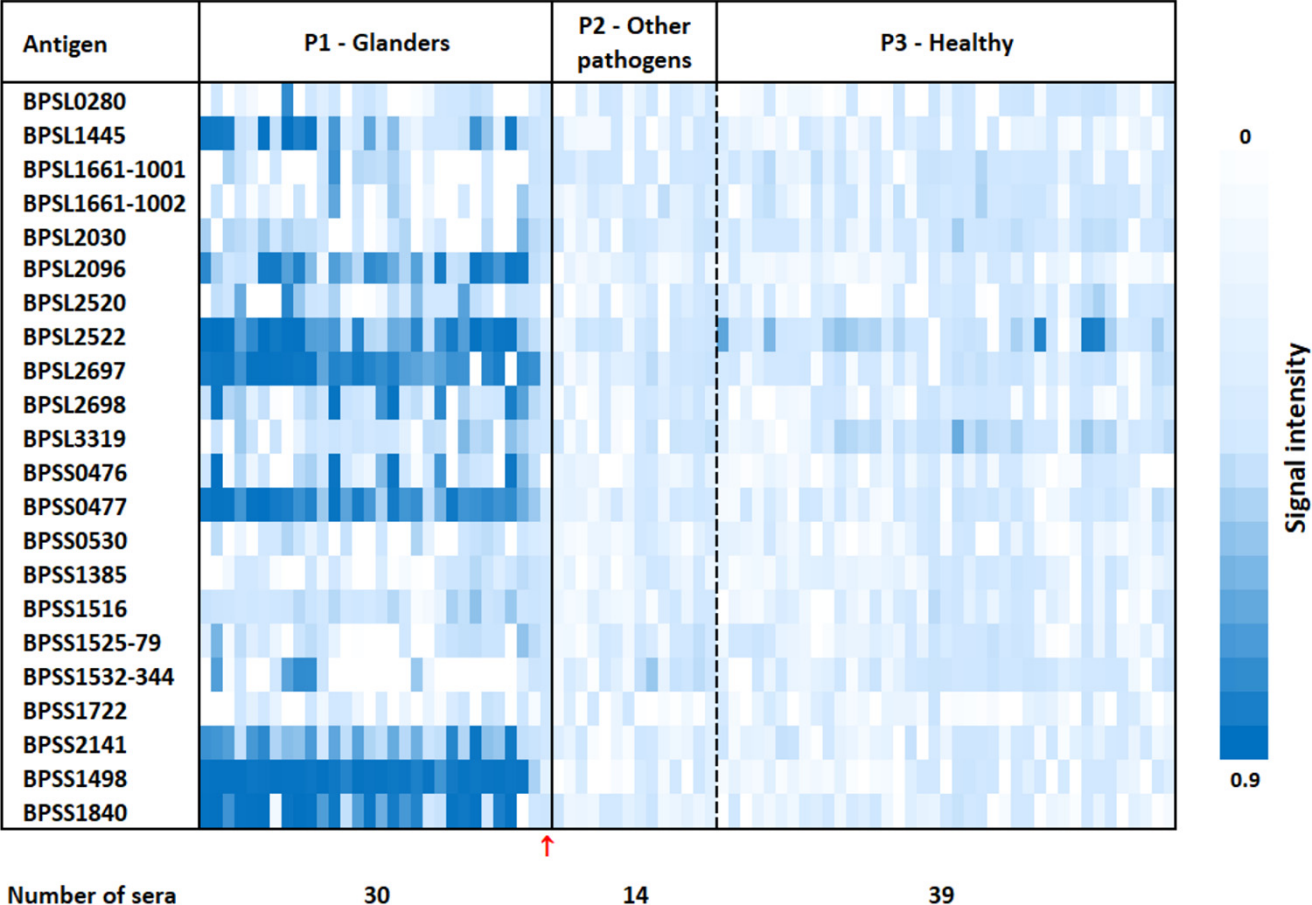
Heatmap showing the IgG response against B. pseudomallei proteins spotted on a microarray probed with glanders sera (panel P1, *n* = 30) and controls (P2, horses infected with other pathogens [*n* = 14], P3, healthy subjects [*n* = 39]). Sample 18-5616_38 from an exposed animal (with close contact to glanders-positive animals during an outbreak) showing negative test results by standard glanders serology tests is indicated by a red arrow.

The multiplex detection by the microarray allows for a detailed analysis of the results in single-antigen resolution to identify the most prominent protein antigen biomarkers for glanders serology. The results are shown in the heat map in Fig. 2 and Table S3. Each antigen row showing a high horse IgG antibody response (dark blue) for the glanders sera (P1) and no or a low response (white to light blue) in the case of controls (P2 and P3), indicates a discriminative antigen for glanders.

The microarray assay achieves a diagnostic sensitivity of 96.7% (confidence interval [CI], 85.5 to 99.6%) and a diagnostic specificity of 100.0% (CI, 95.4 to 100.0%) if at least one of the two antigens, BPSS1498 (Hcp1) or BPSL2697 (molecular chaperone GroEL), gives rise to a signal. Interestingly, the only serum in this study that was included based solely on clinical symptoms and close contact to glanders-positive horses during an outbreak gave a negative test result in our microarray assay. This is in agreement with the other tests applied (see Table S1, panel P1, “18-5616_38”). Hence, it might well be that the animal is true negative and was suffering from a different disease.

All except four (BPSS0530, BPSS1385, BPSS1516, BPSS1722) of the 22 proteins showed signal intensities above the array threshold (≥0.3) for at least one serum sample, indicating their usefulness in multiplex detection.

**FIG 3:**
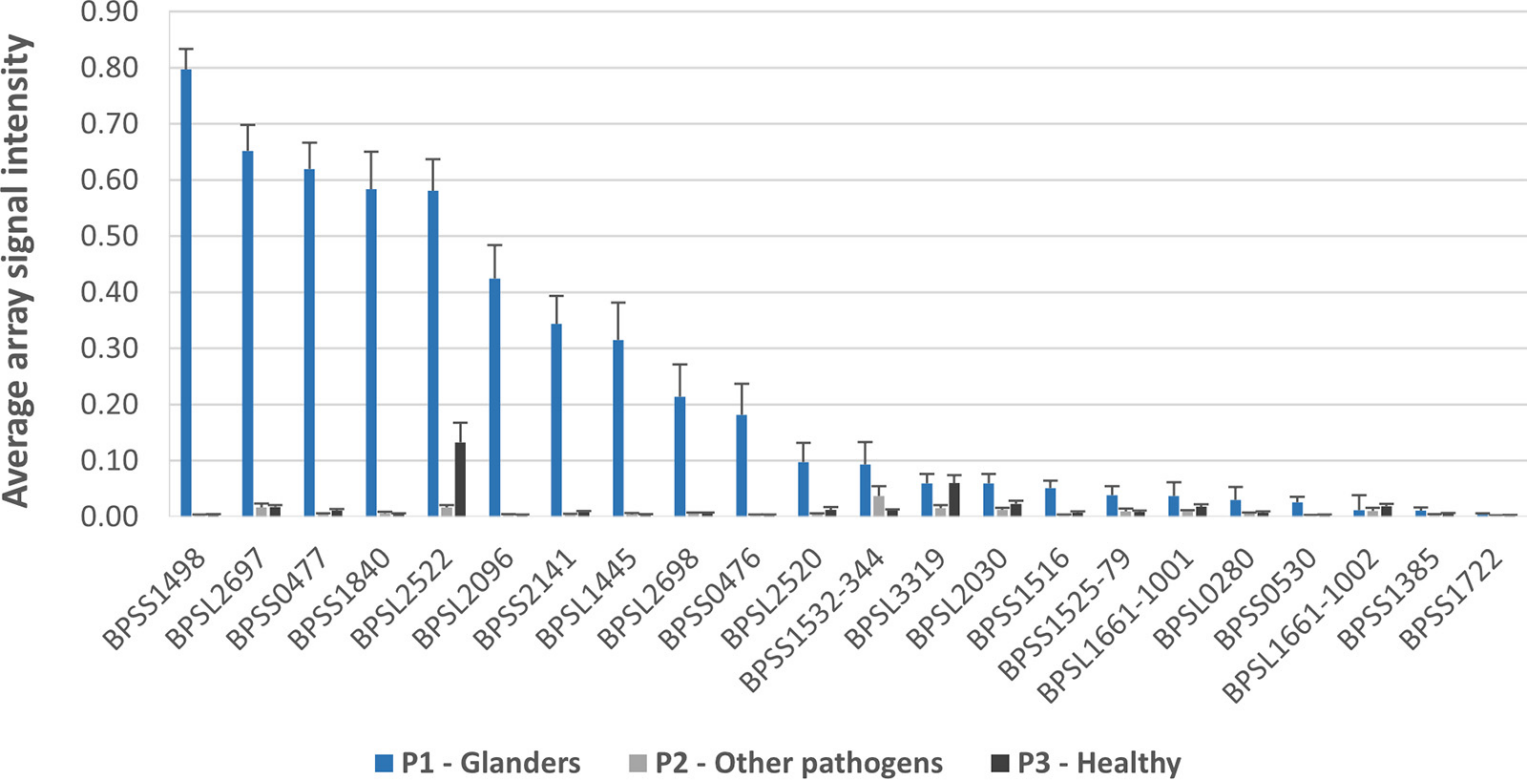
Average B. pseudomallei microarray signal intensities of equine IgG antibodies bound to the indicated protein antigens when incubated with glanders sera and controls. Error bars indicate the standard error of the mean.

As illustrated in Fig. 2 and 3 and confirmed by statistical analysis (Table S4), 12 out of 22 protein antigens (BPSS1498, BPLS2697, BPSS0477, BPSS1840, BPSL2522, BPSL2096, BPSS2141, BPSL1445, BPSL2698, BPSS0476, BPSL2520, BPSS1532) differed significantly between sera from panel P1 and control sera from panels P2 and P3. The highest signal intensities on average were obtained for BPSS1498 (Hcp1), BPSL2697 (molecular chaperone GroEL1), and BPSS0477 (molecular chaperone GroEL2), proteins of interest that have already been mentioned for glanders (17, 19, 20), but also for new proteins described in our study, such as BPSS1840 (putative *N*-acetylmuramoyl-l-alanine amidase), BPSL2522 (outer membrane protein A precursor), and BPSL2096 (putative hydroperoxide reductase).

Interestingly, the seroreactivity of the antigens for glanders and the order of their mean signal intensity are similar but not identical to those observed for melioidosis patients (21). Indeed, among this set of 12 antigens of interest, pronounced differences were observed for BPSS1840 (putative *N*-acetylmuramoyl-l-alanine amidase), BPSS2141 (putative periplasmic oligopeptide-binding protein), and BPSL1445 (putative lipoprotein), for which greater responses were obtained with sera from glanders-infected horses. Future studies should investigate whether these candidates are able to differentiate between the two diseases or if these results are due to, for example, different sample types (e.g., human versus horse sera), different antibody detection systems, and/or stage of the disease.

To the best of our knowledge, no other tool allows the detection of such a high number of serodiagnostic protein antigens for glanders in parallel and, as such, it enables an unprecedented overview of the serologic response in a single and simple experiment. In addition to a simple positive/negative test result, the analysis of antibody response patterns of multiplex assays might allow one to differentiate between glanders and melioidosis and infected and exposed animals, among others.

### Antigen selection and establishment of test conditions for a rapid and robust glanders point-of-care (POC) dipstick test.

Based on the promising antigen candidates identified from our array experiments, we aimed to develop a rapid POC dipstick test for glanders diagnostics. Unlike microarray tests that require comparably expensive special equipment and software for analysis, no special laboratory equipment is required for a POC dipstick test. This could even be further simplified in an on-site setting by providing preportioned master mix and using a simple capillary of the correct volume instead of a pipette for serum dilution. This makes the test ideally suited for a wide range of applications, from routine laboratories to on-site testing. Compared to cassette-based POC assays (e.g., lateral flow format), dozens of dipsticks can be run in parallel in a microtiter plate, facilitating handling and reducing space requirements to an absolute minimum.

We selected the three best antigens (BPSS1498, BPSL2697, BPSS1840) based on the criteria high discriminatory power and average signal intensity (see Fig. 2 and 3) for the development of a multiplex dipstick. As slight sequence differences between B. pseudomallei and B. mallei homologs were observed by *in silico* comparison (Table 1), the following B. mallei homologs were expressed and purified for the dipstick test: BM45_3167 (Hcp1), BM45_901 (molecular chaperone GroEL), and BM45_4262 (putative *N*-acetylmuramoyl-l-alanine amidase).

BPSS0477 (molecular chaperone GroEL 2) encodes for a second GroEL homolog in *B. pseudomallei* that is not present in the *B. mallei* genome, therefore it was not included in addition to the BPSL2697 (GroEL 1) homolog BM45_901.

Gold-conjugated anti-horse IgG was chosen as a secondary antibody, a widely chosen readout for serological glanders assays (13, 17, 28). A schematic representation of the assay procedure using a serum dilution of 1:100, is shown in Fig. 1.

### Evaluation of the POC glanders dipstick test demonstrates a high serodiagnostic performance.

**FIG 4:**
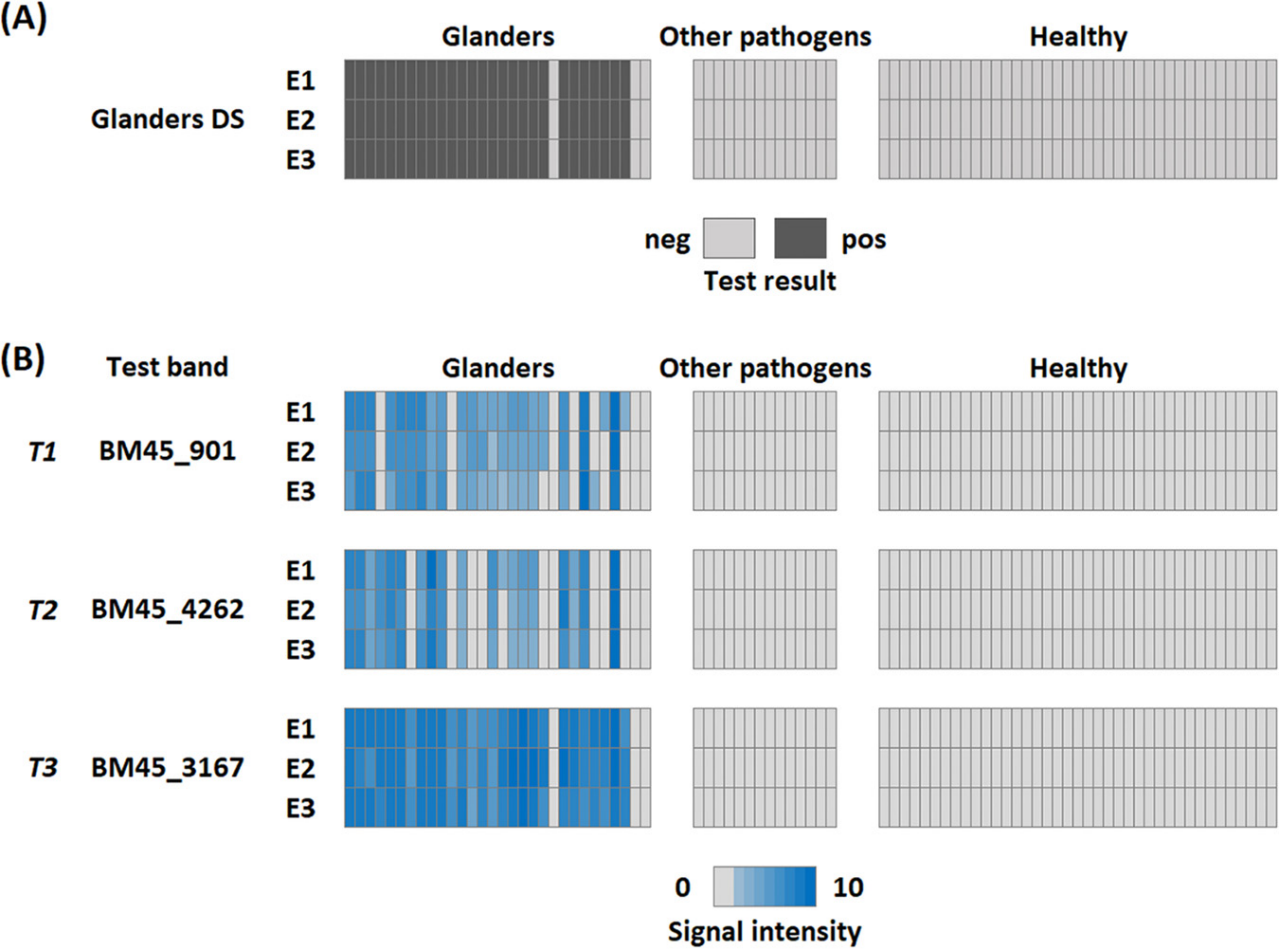
(A and B) Heatmap of POC dipstick results from B. mallei-infected horses and control groups for the overall assay (A) and the three test bands separately (B), both for all three evaluators (E1, E2, E3). The POC dipsticks were considered positive overall if at least one antigen test band gave rise to a signal. A total of 83 sera were tested, including those from confirmed glanders cases (P1, *n* = 30) and controls consisting of sera from horses infected with pathogens than B. mallei (*n* = 14) or healthy horses from glanders-free areas (P3, *n* = 39).

The performance of the glanders POC dipstick test was evaluated with the three serum panels, P1 to P3. The results are summarized in Fig. 4 and Table S5. Overall, our glanders POC dipstick test shows a sensitivity of 90.0% (CI, 75.7 to 97.1%) and a specificity of 100.0% (CI, 95.4 to 100%) in our cohort.

Identical overall results (Fig. 4A) were reported by the three independent evaluators, demonstrating the robustness of the assay and highlighting the remarkable consistency of the results. None of the control sera of panels P2 and P3 were found to be positive by the evaluators. Among all positive sera (panel P1), three sera, including the serum sample 18-5616_38 tested also negative by the microarray, were found negative by all evaluators for all three antigens. Regarding the other sera of this panel, at least one positive response for one antigen was observed by each evaluator. Because the sensitivity of dipstick tests is generally lower than that of microarrays, it is not surprising that two samples found to be initially positive in our microarray test were negative using this rapid test. Both of these sera gave rise to signals of moderate intensity in our array testing (all dipstick antigen homologs below 0.67) and are probably below the detection limit of the dipsticks. It is noteworthy that one of these sera (18-5616_29, P1) was CFT doubtful but iELISA positive, and the other (18-5616_37, P1) was CFT positive but iELISA negative.

Only slight differences in intensity scoring could be observed between evaluators (Fig. 4B). However, no significant difference regarding the presence or absence of a test line between the three evaluators was found (BM45_901, *P* = 0.368; BM45_4262, *P* = 0.368; BM45_3167, *P* = 1.000). Similar to our microarray experiments, all three antigens distinguished significantly between glanders sera and controls for all evaluators (Table S6).

Comparing the results of the three antigen test bands selected, it becomes apparent that protein BM45_3167 (Hcp1) alone might be sufficient for glanders serodiagnostics. However, Hcp1 variants with diminished antigenicity that affects test performance have been reported for human melioidosis patients (29). Moreover, the availability of several antigens makes it possible to consolidate the diagnosis for a regulated disease with important consequences for both animal and human health. Each additional positive antigen test band makes it more unlikely that the test is false-positive, as chances for two or more nonspecific antibodies against the antigens used decrease. Of note, if two proteins have to react for a dipstick to be considered positive, our test still has a sensitivity of 78.9% (CI, 69.6 to 86.3%). Larger prospective studies in areas of endemicity in the future will help to decide on the ideal mode of evaluation of our POC glanders dipstick test and will reevaluate its results in different animal cohorts.

In conclusion, we have used the microarray-based assay developed for the screening of antigens of interest for human melioidosis to successfully identify antigens of interest for glanders in equids. About 10 antigens of interest were identified, and of these, 3 B. mallei variants were used for the development of a rapid, highly parallelizable test, allowing a result in 15 min. The test matches the diagnostic performance of sophisticated glanders assays in a POC format (Table 2). Furthermore, it performs equally well regarding sensitivity and specificity as the serological gold standard for glanders, the CFT. However, it is much more reliable, giving results for 100% of the sera tested, whereas the CFT suffers from anticomplementary and doubtful test results, which only leads to evaluable results in 66.7% of panel P1 (infected animals) in this study. Therefore, the dipstick test presented here is ideally suited as a stand-alone POC test for glanders serodiagnostics.

**TABLE 2:** Sensitivity and specificity of the serodiagnostic tests applied for glanders applied^*a*^

Test	Sensitivity (%) (CI)	Specificity (%) (CI)
Glanders dipstick	90.0 (75.7–97.1)	100.0 (95.4–100.0)
Microarray	96.7 (85.5–99.6)	100.0 (95.4–100.0)
CFT	70.0 (48.3–86.4)	100.0 (95.4–100.0)
iELISA	90.0 (75.7–97.1)	98.0 (91.0–99.8)

a Sera with doubtful or anticomplementary activity in the CFT were excluded for the performance calculations of the CFT. Confidence intervals shown in parenthesis. The difference in sensitivity (*P* = 0.054) and specificity (*P* = 0.392) observed was not significant between all assays.

Our described approach offers prospects for the search of markers of interest in both B. pseudomallei and B. mallei infections in various animal species and, possibly, for different stages of infection (e.g., acute versus asymptomatic) in the sense of a One Health approach.
