# Supplementary material for: Protein Microarray-Guided Development of a Highly Sensitive and Specific Dipstick Assay for Glanders Serodiagnostics
Source: J Clin Microbiol. 2022 Dec 21;61(1):e01234-22. doi: 10.1128/jcm.01234-22 (PMC9879090; doi:10.1128/jcm.01234-22)
Supplement: Supplemental file 7 — Captions to Tables S1 to S6. Download jcm.01234-22-s0007.pdf, PDF file, 0.5 MB [file jcm.01234-22-s0007.pdf]

Supporting Information

Table S1: Serum information including country of origin, infecting pathogen and test results for our assays and common glanders assays (CFT, iELISA).

Table S2: Locus tag, abbreviated name, cloning primers and expression constructs of additional protein antigens used in this study.

Table S3: Protein microarray results from *B. mallei*-infected horses and controls. Antigen spots were considered positive if the signal intensity was greater or equal to 0.3. A total of 83 sera were tested, including glanders-positive sera (n = 30) and controls consisting of sera from horses infected with other pathogens (n = 15) and healthy horses (n = 38).

Table S4: Single antigen sensitivity, specificity and Fisher's exact test results for signal intensity comparison between glanders and control sera on the protein microarray.

Table S5: Dipstick signal intensities and results for samples from *B. mallei*-infected horses and controls. Data is shown for (A) the overall assay and (B) all four protein antigens, both for all three evaluators (E1, E2, E3). Results for the single antigens were obtained by comparing the dipstick bands to the gold reference card (a cutoff for the readout by eye equals 4). Dipsticks were considered generally positive if at least one antigen test band gave rise to a signal. A total of 83 sera were tested, including glanders-positive sera (n = 30) and controls consisting of sera from horses infected with other pathogens (n = 15) and healthy horses (n = 38).

Table S6: Fisher's exact test results for single antigen test bands distinguishing between glanders and control sera in the dipstick assay.
